# Research and Remedies

**Published:** 1912-09-21

**Authors:** 


### RESEARCH AND REMEDIES.

### A New Clinical Test for Syphilis.

A cutaneous reaction, on the model of those employed for tuberculosis, has been devised by Noguchi for syphilis; and Wolfsohn, who has independently tested it, describes the method and its results in the *Johns Hopkins Hospital Bulletin.* A (sterile) culture of the Treponema pallidum, prepared in a special way and termed *luetin*, is injected intradermally over the biceps muscle of the left arm; a similar quantity of the culture medium, but without any micro-organisms, is injected in a similar position into the right arm. If nothing further develops than a slight blush, a moderate induration, or a papule, disappearing within forty-eight hours, the reaction is negative. A positive reaction is indicated by a large indurated papule on an erythematous base, or by a group of vesicles on an indurated tender base, or by pustule formation. These manifestations may occur within twenty-four hours, or they may be delayed for many days; they are said to be easily distinguishable from the very slight lesions which constitute a negative reaction. The luetin reaction is believed to be specific for syphilis; that is, is never found in non-syphilitic cases. It is of greatest value in the latent and tertiary stages of syphilis. In parasyphilitics with cardiovascular manifestations the reaction may be delayed up to thirty days. These conclusions are based upon an experience of one hundred and fifty cases.

### Orthostatic Albuminuria.

This puzzling condition of albuminuria in adolescents or young adults still affords many problems for solution in respect of prognosis, diagnosis, and treatment. The ætiology, too, is still obscure, though several hypotheses have been put forward with a certain amount of plausibility. In the *Medical Review* the views of Jehle and Hamburger are quoted at some length as to this aspect of the condition. The former attributes orthostatic albuminuria to lordosis of the spine in the lumbar region; mechanical stasis in the kidneys was supposed to be thus induced. The objection to this idea is that many patients with marked lordosis do not get albuminuria, and that those who do get it exhibit it only temporarily as a rule. Hamburger holds that there is in addition a vasomotor or angioneurotic element; the patients often exhibit instability of the vasomotor system, and puberty is especially the age for both these phenomena and those of orthostatic albuminuria. He quotes the case of a boy of thirteen who was suffering from neurosis as a result of a "purely psychic trauma." The albuminuria contained no albumin at first; but after extreme lordosis had been maintained in a kneeling position for five minutes, nausea and vomiting came on and the urine was solid on boiling. A few days later, when treatment had restored the boy to health, the lordosis of Jehle's position caused only a trace of albumin and no nausea. Hamburger subsequently constructed an apparatus for holding a child in a position of lordosis and for gauging the degree of the latter. He found that under exactly uniform conditions the same patient, when subjected to this experiment, excreted sometimes as much as 0.4 per cent. of albumin and at others as little as 0.01 per cent. He concludes that the mechanical effect of lordosis is the prime cause of this species of albuminuria, and that vasomotor instability is a subsidiary factor.

### The Newest Serum Therapy.

Not long ago the announcement was made that a foreign observer was prepared to diagnose the existence of pregnancy by means of a reaction in the blood. It is true that not much has been heard of the idea since, but for all that it is not at all an impossibility. The latest development, however, announced from America and from Germany, is the discovery of a serum which is capable of bringing about discharge of the ovum from the pregnant uterus. Since it is claimed that this result will follow the use of the serum at any stage of pregnancy, it is obvious that (provided investigation confirms the claims made) the manufacture and use of this serum will have to be very carefully supervised. Indeed we do not know of any more convincing argument in favour of the views expressed in The Hospital (June 1, p. 214) as to the desirability of Government intervention in the serum and vaccine industry.

The rationale of the process is this. It is thought possible that the cause which determines the expulsion of the fœtus at the termination of a normal pregnancy is the accumulation of some substance in the fœtal blood which acts as an oxytocic. Therefore, after a normal delivery of a healthy child, the cord is cut with a sterile instrument and the blood which drips from the placental end is collected in a sterile vessel. The serum is then separated from this blood, filtered through a very stringent filter said to arrest all micro-organisms, and stored on ice for a month. If at the end of this time a portion of it shows no growth when inoculated into media, the serum is pronounced fit for use. This interval of a month is further useful, in that it enables the infant to be watched for any signs of constitutional taint such as would disqualify its serum. To induce labour, say in a case of contracted pelvis, all that is necessary is to inject 2 to 20 c.c. of this serum; and uterine contractions will, it is stated, commence within twenty-four hours. The two objections which strike one *a priori* are: first, the risk of transferring the infection of syphilis or other disease; and, secondly, the unfairness to the infant of cutting the cord before the circulation in it has entirely ceased. We understand that arrangements are being made for an independent test of the method in London. It is to be hoped that the results, whether favourable or unfavourable, will be published in due course. An able account of the work done abroad is given by Rongy in the July number of the *American Journal of Obstetrics and Gynæcology.*

